# Bioinformatics revealed biomarkers for diagnosis in kidney stones

**DOI:** 10.3389/fgene.2025.1542840

**Published:** 2025-03-18

**Authors:** Ziqi He, Chao Song, Zhong Wang, Caitao Dong, Qinhong Jiang, Xi Yu, Guang Shan

**Affiliations:** ^1^ Department of Urology, Renmin Hospital of Wuhan University, Wuhan, China; ^2^ Department of Breast and Thyroid Surgery, Renmin Hospital of Wuhan University, Wuhan, Hubei, China; ^3^ Department of Anesthesiology, Renmin Hospital of Wuhan University, Wuhan, Hubei, China

**Keywords:** bioinformatics, kidney stone, diagnostic biomarkers, ferroptosis, immune microenvironment

## Abstract

**Background:**

One of the most prevalent urinary illnesses is kidney stone formation, often known as nephrolithiasis. The precise processes of kidney stone remain poorly known after substantial investigation. In order to successfully prevent and cure stone formation and recurrence, additional research into the pathophysiology of stone formation is of paramount importance. Ferroptosis is linked to a variety of renal diseases and is a critical factor in the death of cells. However, little is known about how ferroptosis-related genes (FRGs) contribute to the development of kidney stones.

**Methods:**

The Ferroptosis Database and the Gene Expression Omnibus (GEO) database provided us with information on kidney stones and FRGs, respectively (FerrDb).

**Results:**

Eight DE-FRGs related to kidney stones were found in total, and they were all closely related to immune response and autophagy management. Following this, among the 8 DE-FRGs, LASSO and SVM-RFE algorithms chose FZD7, STK11, SUV39H1, and LCN2 as marker genes with suitable diagnostic capabilities. These marker genes may be involved in the control of the PPAR signaling pathway, mTOR signaling system, and fatty acid production of kidney stones, according to the functional enrichment analysis that followed. Additionally, 24 drugs that target two marker genes have been found. Despite this, the ceRNA networks have gained that the regulatory relationship between marker genes is rather complex. Additionally, the findings of the CIBERSORT investigation indicated that FZD7 and SUV39H1 may be linked to variations in the immune milieu of people who have kidney stones.

**Conclusion:**

We developed a diagnostic tool and provided information on the development of kidney stones. In order to confirm its diagnostic applicability for kidney stones, more studies are needed before it may be used in clinical practice.

## 1 Introduction

One of the most prevalent urinary illnesses is kidney stone formation, often known as nephrolithiasis (Khan et al., 2016). High frequency, high recurrence rates, and high treatment costs for kidney stone have a detrimental effect on both individuals and society (Yang et al., 2020; Li et al., 2019). Kidney stone, which may cause significant back pain and potentially lead to serious consequences including acute renal failure, acute kidney injury, has recently caused great worry around the globe. If proper precautions are not implemented, the recurrence rate for individuals who have had stone removal treatment is probably between 35% and 50% (Albert et al., 2020). The precise processes of kidney stone remain poorly known after substantial investigation. The development of particular targeted medicines has been severely constrained precisely because relatively limited progress in explaining the process has been made. Therefore, in order to successfully prevent and cure stone formation and recurrence, additional research into the precise pathophysiology of stone formation is of paramount importance.

Ferroptosis, a novel and uncommon kind of cell death, has just been found. Ferroptosis is an iron-dependent type of cell death, as opposed to apoptosis, pyroptosis, and receptor-interacting protein kinase-dependent necroptosis (Dixon et al., 2012). The key mediators of ferroptosis are the antioxidant enzyme glutathione peroxidase 4 (GPX4) and the cystine/glutamate antiporter system Xc^−^ (xCT). When there are insufficient quantities of xCT and GPX4, intracellular cystine levels fall. This, in turn, reduces the quantity of glutathione that can be produced and slows the rate at which lipid peroxide can be broken down. Ferroptosis, which is brought on by a buildup of lipid peroxide that takes place on the interior of the cell, is the consequence of both of these alterations having taken place (Dixon and Stockwell, 2014; Yang and Stockwell, 2016; Gaschler and Stockwell, 2017). Recent studies have shown that ferroptosis contributes to the death of tubular cells in acute renal damage (AKI) (Yang and Stockwell, 2016; Louandre et al., 2013). The small-molecule medication known as ferrostatin-1 (Fer-1), which reduces lipid oxidation and, as a consequence, ferroptosis, may reduce the degree of tube damage in experimental models of AKI (Martin-Sanchez et al., 2017; Linkermann et al., 2014). It was shown that GPX4 in tubing cells has a renoprotective function, as proteinuria, capillary edema, and tubing cell death were dramatically elevated in GPX4-deficient animals. On the other hand, in contradiction to AKI, very little is known about the role that ferroptosis plays in the formation of kidney stones.

As a result, we used bioinformatics research to investigate genes linked to ferroptosis in the hope of identifying possible kidney stone biomarkers. In addition, we investigated the role that these genes play in the production of kidney stones.

## 2 Methods

### 2.1 Data source

In the course of this research, queries were made to the GEO database in search of information on the gene expression of normal papillary tissue from calcium stone former, and normal papillary tissue from control patients without any kidney stone. There were a total of 62 samples in the GSE73680 collection, 29 of which were normal papillary tissue from calcium stone forme, and 33 of which were normal samples. This data collection is being used as a training set for the analysis that will be carried out in the primary portion of the study. The FRGs that were employed in this investigation totaled 728 and were obtained from FerrDb. Supplementary Table S1 shows the information about the genes. We used the Drug Gene Interaction Database (DGIdb) to make a prediction about the likelihood of medications targeting marker genes. In addition to that, the DrugBank database was mined for information on the chosen pharmaceuticals' structural makeup.

### 2.2 Differential expression analysis and functional enrichment analysis of FRGs

We initially collected expression information for 728 FRGs (only 439 FRGs were expressed in this dataset) from the GSE73680 collection for normal samples and kidney stone samples. Then, using R’s Student’s t-test, the FRGs with different expression between the normal and kidney stone samples were found. Genes with p-value below 0.05 were deemed to be significant. In the clusterProfiler program, the Kyoto Encyclopedia of Genes and Genomes (KEGG) and Gene Ontology (GO) are extensively accessible (Yu et al., 2012; Kuleshov et al., 2016). The biological features of the genes and genomes of all species are characterized by the three sub-ontologies of the GO annotation. These sub-ontologies are known as biological processes (BPs), cellular components (CCs), and molecular functions (MFs) (Gene Ontology, 2006). Statistics were deemed to be significant when the p-value was lowered to less than 0.05.

### 2.3 Finding the best gene biomarkers for diagnosing kidney stone

In order to lessen the amount of space used by the data, the least absolute shrinkage and selection operator (LASSO) method was implemented using the glmnet software package (Yang et al., 2019; Friedman et al., 2010). In order to assist feature selection and the discovery of gene biomarkers associated with kidney stones, DE-FRGs were maintained between kidney stone patient samples and normal samples. In the meanwhile, a contrast was drawn between the error rates generated by the 5-fold cross-validation of the support vector machine-recursive feature elimination (SVM-RFE) model and the error rates generated by the support vector machine-recursive feature elimination (SVM-RFE) model (Qiu et al., 2017). In addition, the overlapping biomarkers generated by the two distinct approaches were used to identify the most effective gene biomarkers linked with kidney stones. Utilizing the receiver operating characteristic (ROC) curve, the diagnostic performance of the top gene biomarkers was evaluated. This was followed by the computation of the area under the curve (AUC) in addition to the precision, sensitivity as well as specificity. In addition, the glm R package was used to create a logistic regression model on the basis of seven marker genes, which can forecast the different types of sample in GSE73680 dataset. This model was developed to anticipate the outcomes of the preceding stage. Utilizing ROC curves, more study was conducted to determine the diagnosis accuracy in the logistic regression model.

### 2.4 Enrichment analysis using gene set enrichment analysis (GSEA) and gene set variation analysis (GSVA)

In order to carry out this study, the GSEA R package was used (V.4.1.0). In order to investigate the related pathways of the seven marker genes more deeply, the connection between the marker genes and every other gene included in the GSE73680 dataset was analyzed. The results of sorting all of the genes from highest to lowest correlation were then used in the study, and the gene set that was produced was taken into account. The KEGG signaling pathway set was utilized as a preset in the interim so that the enrichment of the gene set could be established. KEGG stands for the Kyoto Encyclopedia of Genes and Genomes. The GSVA R package was used in the carrying out of this inquiry (V.1.38.0). Examination of the variance in gene sets (GSVA) (Hanzelmann et al., 2013). In this investigation, an individual GSVA analysis was performed on each marker gene, with the KEGG pathway set serving as a background gene set. In parallel, we used the limma tool to investigate the disparity in GSVA score that existed between the high-expression and low-expression groups of the marker gene. |t| was more than two under the screening circumstances, and the p-value was less than 0.05. If t was more than zero, we assumed that the route was active in the group that had a high expression, and if t was less than zero, we assumed that the pathway was active in the group that had a low expression.

### 2.5 Immune infiltration analysis of marker genes and construction of the ceRNA network

The CIBERSORT technique analyzes the patterns of gene expression seen in complex tissues in order to estimate the cell composition of these tissues (Newman et al., 2015). Using the CIBESORT tool, we analyzed the data from the GSE73680 dataset to determine the percentage of each of 22 distinct kinds of invasive immune cell types present in each tissue. The total number of immune cell type fractions that were evaluated for each sample was 1, which equaled the sum of all of the evaluations (Zhang et al., 2019). The four marker genes were used as the input for a prediction of mRNA-miRNA interaction pairs that were produced using the starBase database. During this interim period, the RNA sequences of four marker genes were found in the National Center for Biotechnology Information, and the human microRNA sequences could be downloaded from the miRbase database. Both of these sequences were collected from the National Center for Biotechnology Information (NCBI). Following the Miranda algorithm’s prediction on the possible pairing of mRNA and miRNA nucleic acid, the threshold for what constitutes a successful binding score was increased to 170. (The number 140 served as the starting point.) Following that, in order to get the mRNA-miRNA-lncRNA ceRNA network, we searched starBase for predicted miRNA and screened miRNA-lncRNA.

### 2.6 Build the oxalate-induced model and cell culture

We obtained human proximal tubular epithelial cells (HK-2) for our study from Shanghai, China’s Cell Bank of the Chinese Akidney stoneemy of Sciences. At 37°C and 5% carbon dioxide, HK-2 cells were grown in Dulbecco’s Modified Eagle Medium (DMEM/F12, Gibco, China) with 10% foetal bovine serum (FBS, Biological Industries, Israel) and 1% penicillin/streptomycin (C0222, Beyotime Biotech Inc, Shanghai, China). The culture medium was changed every day, and the cells were passed, when the cell density reached 70%–80% confluence. We created a model of oxalate-induced cell injury using 2 mM oxalate (75,688, Sigma, Germany) for 12 h, much as in our prior study.

### 2.7 Reverse transcription polymerase chain reaction in real time (qRT-PCR)

TRIzol reagent (15596026, ThermoFisher, United States) was used to extract the total RNA from the treated cells in accordance with the manufacturer’s instructions. The Hifair^®^ III first Strand cDNA Synthesis Kit (gDNA digester plus) and 2 L of RNA were used to create the cDNA (11139ES60, Yeasen Biotechnology Co., Ltd., Shanghai, China). Then, using a LightCycler480 (Roche Diagnostics, United States), quantitative real-time PCR was carried out using Hieff UNICON^®^ Universal Blue qPCR SYBR Green Master Mix (11184ES08, Yeasen Biotechnology Co., Ltd., Shanghai, China). GAPDH was utilized as an internal standard control, and the relative expression fold changes were calculated using 2^−ΔΔCT^ methods. Three copies of each qRT-PCR experiment were performed. In order to conduct real-time PCR, the following primer sequences were used: GAPDH forward: 5′-GGA​GTC​CAC​TGG​CGT​CTT​CA-3'; reverse: 5′-GTC​ATG​AGT​CCT​TCC​ACG​ATA​CC-3′.

FZD7 forward: 5′- CTA​CCG​CGC​CCT​ACC​TG-3'; reverse: 5′- AAA​GTA​CAT​CAG​GCC​GTT​GG-3′.

SUV39H1 forward: 5′- GAG​TCG​CCT​GAA​ATG​ACA​GA-3'; reverse: 5′- GCA​CAC​TGG​GAA​ACG​CT-3′.

STK11 forward: 5′- CTGGACTCGGACGCT-3'; reverse: 5′- AAT​ATT​CCT​GTT​CGC​GGA​TCT-3′.

LCN2 forward: 5′- TCA​CCC​TCT​ACG​GGA​GAA​CC-3'; reverse: 5′- GGT​CGA​TTG​GGA​CAG​GGA​AG-3′.

### 2.8 Animal experiment

Our animal experiment protocols were approved by the Laboratory Animal Welfare and Ethics Committee of Renmin Hospital of Wuhan University. A total of 10 five-week-old male Sprague–Dawley (SD) rats (130–180 g) were used in the experiments. The rats were randomly divided into two groups (n = 5 in each group), as follows: the control group was fed normal drinking water and feed; the stone model group was fed drinking water containing 0.75% EG and normal feed. After 4 weeks of treatment, the rats were euthanized with sodium pentobarbital (30 mg/kg) and carbon dioxide induction. Airflow was regulated with a carbon dioxide concentration between 30% and 70% V/min for about 1 minute, followed by a minimum of 1 minute of air circulation post-clinical death to prevent reversal. And kidney tissues were removed and collected for further analysis.

### 2.9 Data analysis

The Student’s t-test was used to do the comparison between the two groups. With the use of Pearson’s correlation analysis, it was feasible to establish a relationship between 8 different DE-FRGs. The Jvenn program was used to make the Venn diagram that you see here. Through the use of the application Cytoscape, the ceRNA network was shown. If p is less than 0.05, then it is significant. Using R, we were able to finish all of the analyses.

## 3 Results

### 3.1 Identification of DE-FRGs in the GSE73680 dataset

**FIGURE 1:**
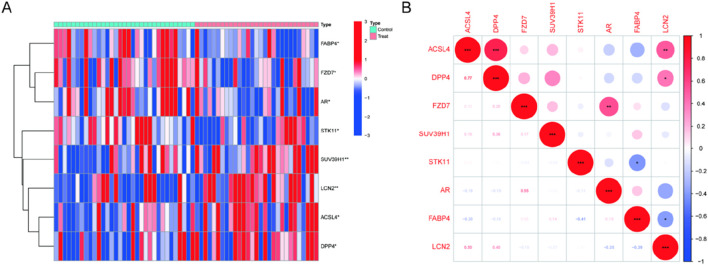
Expression levels of DE-FRGs in kidney stones. **(A)** Heatmap plots illustrate DE-FRG expression patterns across samples. **(B)** The relationship between these genes.

8 of 237 ferroptosis-related genes (FRGs) showed significant differential expression, with 4 genes upregulated and 4 genes downregulated in Randall’s Plaque tissue from calcium stone formers compared to normal papillary tissue from control patients without kidney stones (Supplementary Table S2). The clustering heatmap displayed the samples' DE-FRG expression pattern (Figure 1A). Figure 1B showed the association between these genes. DPP4 and LCN2 were positively correlated with ACSL4. Surprisingly, there was no correlation between SUV39H1 and any DE-FRGs.

### 3.2 Analyses of the DE-FRGs' functionality

**FIGURE 2:**
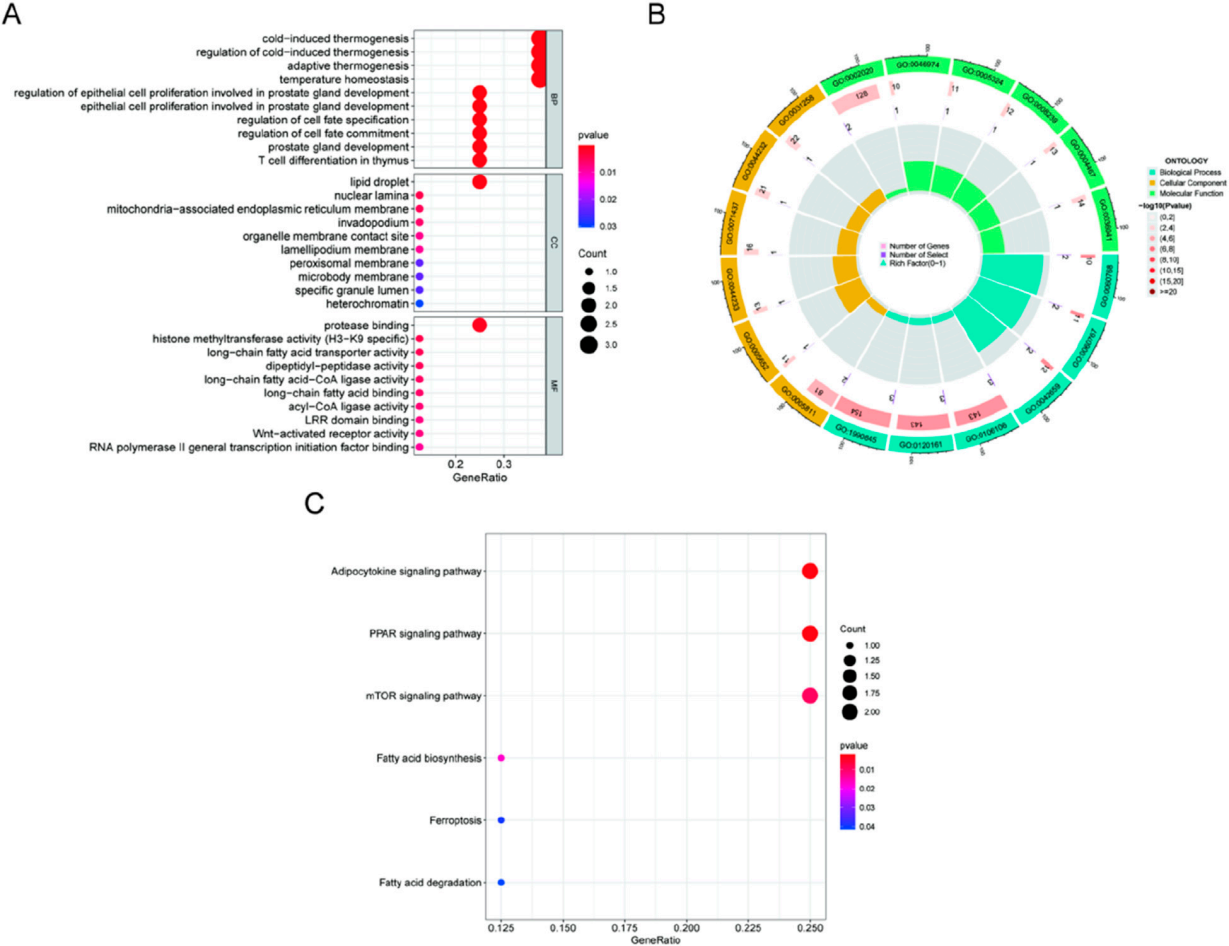
Analyses of functional enrichment for DE-FRGs. **(A, B)** DE-FRG enrichment analysis using GO. **(C)** Enrichment analysis of immunological characteristic gene sets using the KEGG database.

GO enrichment and KEGG pathway analysis were carried out to clarify the biological processes and pathways connected to the DE-FRGs. As a result, GO enrichment analysis revealed that the DE-FRGs are significantly enriched in biological processes related to lipid droplet formation and protease binding (Figures 2A, B). KEGG pathway analysis revealed significant enrichment of pathways related to adipocytokine signaling, PPAR signaling, mTOR signaling, and fatty acid biosynthesis (Figure 2C). Due to their involvement in the control of the adipocytokine signaling route, PPAR signaling pathway, mTOR signaling pathway, and fatty acid production, DE-FRGs may contribute to the etiology of kidney stones.

### 3.3 4 DE-FRGs were shown to be kidney stone diagnostic genes

**FIGURE 3:**
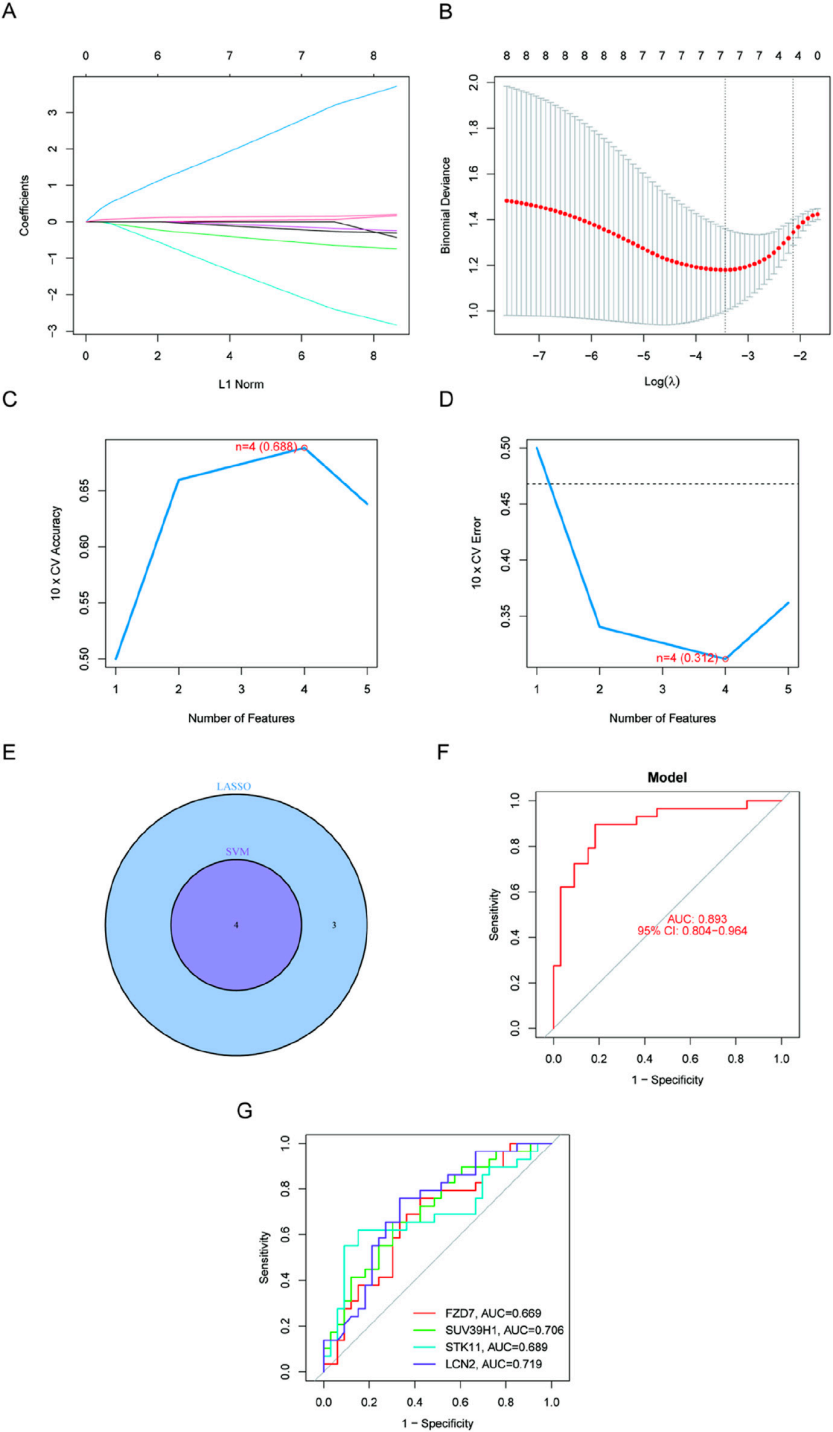
Four DE-FGs have been discovered as kidney stone diagnostic genes. **(A, B)** Seven CAD-related characteristics were chosen using the LASSO logistic regression technique, with penalty parameter adjustment undertaken using 10-fold cross-validation. **(C, D)** SVM-RFE method to filter the seven DE-FRGs to discover the ideal feature gene combination. **(E)** The marker genes derived from LASSO and SVM-RFE models. **(F)** Model using logistic regression to determine the AUC of illness samples **(G)**. ROC curves for each of the four marker genes.

We wished to test the diagnostic efficacy of DE-FRGs by examining the differences between kidney stone sufferers and healthy individuals. Then, to identify kidney stone patients in otherwise healthy individuals, we screened the significant DE-FRGs using the GSE73680 dataset and two separate machine learning algorithms, the LASSO and the SVM-RFE. The LASSO regression analysis approach was used to identify seven kidney stone-related characteristics, and 10-fold cross-validation was utilized to modify the penalty value (Figures 3A, B). The SVM-RFE method was then used to screen the eight DE-FRGs so that the best combination of feature genes could be discovered. Ultimately, it was determined that the optimal feature genes included four genes (the greatest accuracy was 0.688, and the smallest RMSE was 0.312). (Figures 3C, D). When the marker genes derived from the LASSO model and the SVM-RFE model were intersecting, four marker genes (LCN2, SUV39H1, STK11, and FZD7) were chosen for further study (Figure 3E). The succeeding ROC curves showed that the logistic regression model on the basis of four marker genes distinguished normal specimens from kidney stone specimens with an AUC of 0.893% (95% confidence interval: 0.804–0.963). This model was built on the seven previously described marker genes (Figure 3F). In addition, ROC curves were generated for each of the four marker genes to examine how efficiently each gene can distinguish kidney stones from non-stone-affected samples. This was completed in order to respond to the question asked before. Figure 3G demonstrates that the AUC for each gene was more than 0.65. According to the aforementioned results, The logistic regression model, based on the four marker genes, achieved an AUC of 0.893 (95% CI: 0.804–0.963), demonstrating superior diagnostic accuracy compared to individual marker genes.

### 3.4 Marker genes were tightly associated to several pathways that were relevant to kidney stones

**FIGURE 4:**
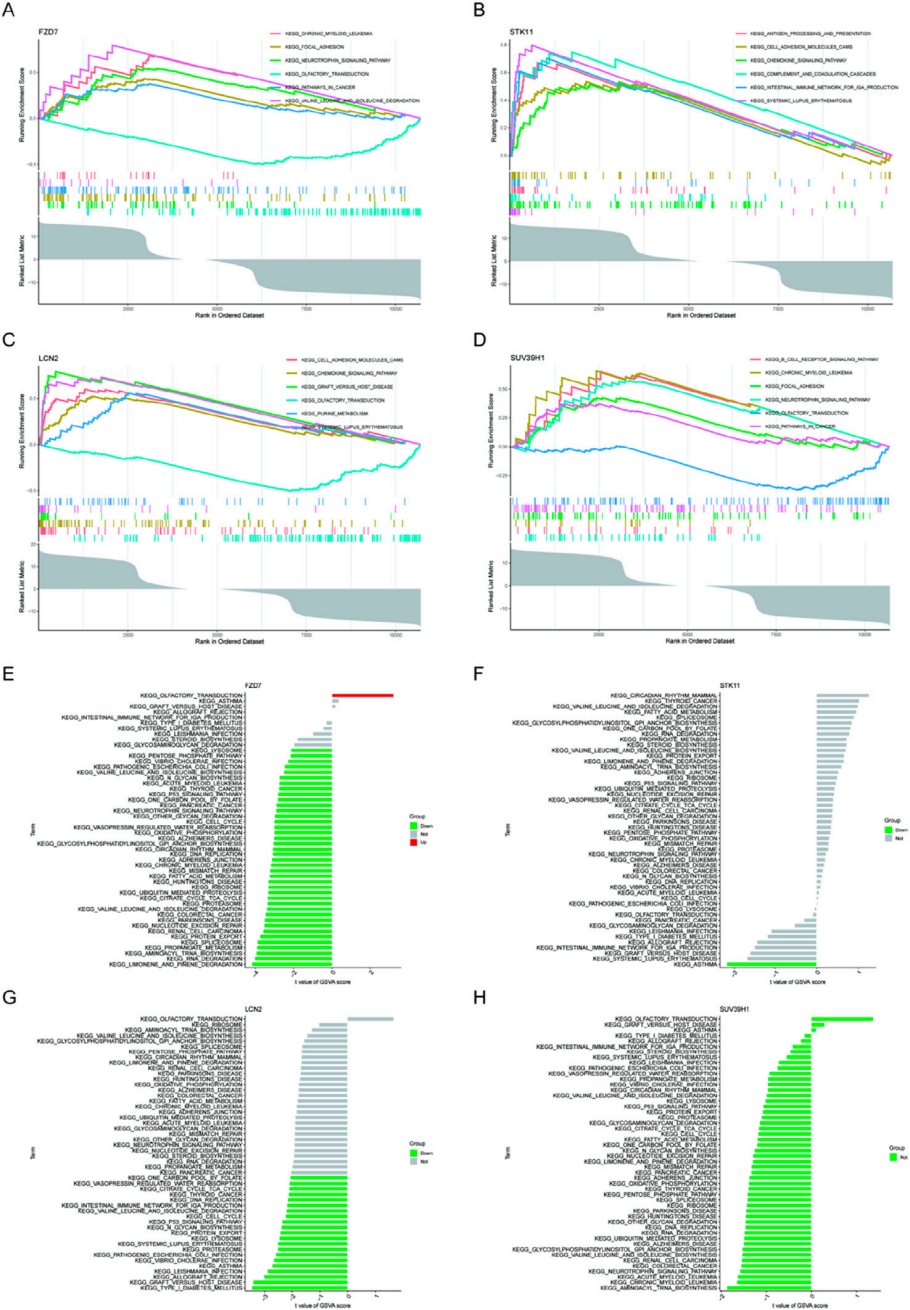
GSEA of KEGG pathway analysis and GSVA analysis of high- and low-expression groups in kidney stones based on the expression levels of each marker gene (www.kegg.jp/kegg/kegg1.html). **(A, E)** FZD7 **(B, F)** STK11 **(C, G)** LCN2 **(D, H)** SUV39H1.

We performed a single-gene GSEA-KEGG pathway analysis to further examine the possible role of marker genes to identify normal papillary tissue from calcium stone former, and normal papillary tissue from control patients without any kidney stone. Figures 4A–D shows the top six pathways that are enriched for each marker gene. Following a thorough investigation, we discovered that the signaling pathways for focal adhesion, chronic myeloid leukemia, and neurotrophin were enriched in FZD7. B cell receptor signaling pathway, chronic myeloid leukemia, and focal adhesion were all enriched in SUV39H1. Additionally, we discovered that STK11 was abundant in chemokine signaling pathway, cell adhesion molecules, and antigen processing and presentation. In addition, we discovered that LCN2 was tightly associated with chemokine signaling pathway, graft versus host disease, and cell adhesion molecules called CAMS. Then, using the GSVA and each marker gene’s expression levels together, we looked at the differentially active pathways between the groups with high and low expression. The findings demonstrated that high levels of FZD7 in the illness may cause kidney stones by triggering olfactory transduction, while low levels of FZD7 triggered limonene and pinene breakdown, and low levels of STK11 were associated with asthma. Type I diabetes was made more active by the downregulation of LCN2. Between high and low SUV39H1 expression, no meaningful route was activated, nevertheless (Figures 4E–H).

### 3.5 Immune landscape research

**FIGURE 5:**
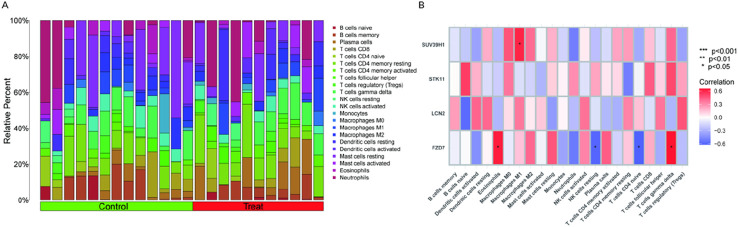
Immune landscape examination. **(A)** Utilized the CIBERSORT method to investigate the immune microenvironment variations between kidney stone patients and normal samples. **(B)** Analysis of the Pearson association between marker genes and immune cell infiltration. (*p < 0.05, **p < 0.01, ***p < 0.001).

The earlier findings suggested a tight connection between the marker genes and the immune response. The immunological microenvironment and kidney stones are inextricably linked, according to a growing body of data. In order to investigate the variations of kidney stone samples compared with normal samples in terms of immune microenvironment, we applied the CIBERSORT method (Figure 5A). Furthermore, Pearson correlation analysis showed that FZD7 showed significant positive correlations with T gamma delta cells (r = X, p < 0.05) and eosinophils (r = X, p < 0.05), and significant negative correlations with NK resting cells (r = X, p < 0.05) and CD4^+^ naive T cells (r = X, p < 0.05). SUV39H1 and M1 macrophages showed a favorable correlation (Figure 5B). These data suggested a potential relationship between FZD7 and SUV39H1 and alterations in kidney stone samples in terms of the immune microenvironmen.

### 3.6 Prediction of medicines that target marker genes

**FIGURE 6:**
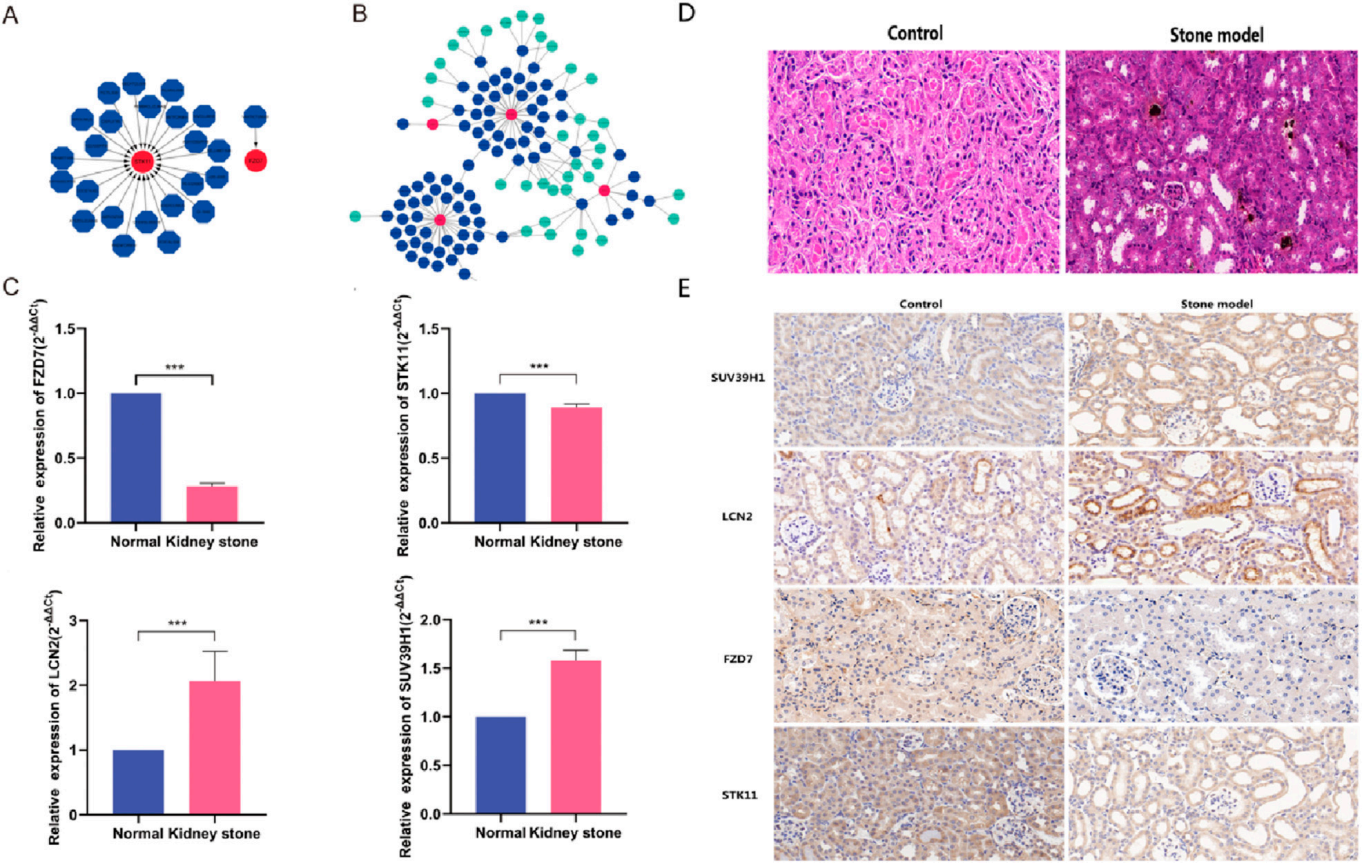
Gene-targeted drug network and marker gene ceRNA network construction. **(A)** Prediction of medicines that target marker genes. **(B)** A network of ceRNAs based on marker genes. **(C)** RNA expression of the marker gene in kidney stone model treated with normal HK2 cells and oxalic acid. **(D)** The silver nitrate staining was used to detect calcium salt deposition and brown block mass indicates calcium salt deposition (magnification, ×400). **(E)** Expression of the marker gene in kidney stone model *in vivo* by IHC (magnification, ×400).

Using the DGIdb database, we also identified potential therapeutic targets for marker genes and examined the interaction between the two parameters when they were left at their default settings. The outcomes that were analysed using the Cytoscape program were displayed in (Figure 6A). Using the DGIdb database, we identified 24 potential drug candidates targeting the marker genes, including 23 for STK11 and one for FZD7, based on *in silico* predictions. Unfortunately, we could not foresee the medications that target SUV39H1 and LCN2.Then, using the starBase and miranda databases, we built a ceRNA network based on four marker genes. The network had 136 edges and 124 nodes (4 marker genes, 81 miRNAs, and 39 lncRNAs) (Figure 6B).

### 3.7 Expression of the marker gene in the kidney stone model *in vitro* and *in vivo*

In the end, the expression level of marker genes in HK2 cells from the kidney model were checked. The expression trends of the GSE73680 dataset were found to match those of FZD7, SUV39H1, STK11, and LCN2. Among these, the expression of SUV39H1 and LCN2 was higher in the kidney stone model than in normal samples (p < 0.001), but the expression of FZD7 and STK11 was lower in the kidney stone model (p < 0.001) (Figure 6C). Meanwhile, we constructed a mouse kidney stone model to detect the expression level of marker genes *in vivo* (Figure 6D). Consistent with *in vitro* results, the expression of SUV39H1 and LCN2 was higher and the expression of FZD7 and STK11 was lower in the kidney stone model than in normal samples (p < 0.001) (Figure 6E).

## 4 Discussion

Ferroptosis, characterized by iron-dependent lipid peroxidation, has been implicated in the pathogenesis of various cancers and diseases affecting the reproductive, neurological, respiratory, and circulatory systems (Wang et al., 2019; Masaldan et al., 2019; Alvarez et al., 2017). But research on the role of ferroptosis in problems with the urinary system has mostly focused on renal failure and renal cancers (Tang and Xiao, 2020; Miess et al., 2018). Few research study how kidney stones are formed or how the symptoms of people with kidney stones deteriorate. It is vital to note that no research has been undertaken on the association between ferroptosis and coronary atherosclerosis. Consequently, it is crucial to select genome - wide chip data, analyze gene expression profiles for two different types of disease, enrich solitary analyses, and enrich metagenomic techniques, such as sharing common ground, analyzing biological variations between kidney stone and regular ferroptosis, and conducting an investigation the molecular pathogenic mechanisms of kidney stone ferroptosis.

Utilizing several gene samples and a substantial quantity of microarray data increases the experiment’s reliability and decreases the error rate. This is a helpful resource for kidney stone therapy and prevention. This page identifies and describes several gene chips that are appropriate for this study. Eight out of 237 FRGs were substantially differently expressed in normal papillary tissue from calcium stone former, and normal papillary tissue from control patients without any kidney stone. This comprises four genes that are upregulated and four genes that are downregulated in compared to normal samples. This group included eight FRGs designated as ACSL4, DPP4, FZD7, SUV39H1, STK11, AR, and FABP4. Differentially expressed ferroptosis-related genes (DE-FRGs) were significantly associated with lipid droplet formation and protease binding activities, according to the findings of an investigation of GO enrichment. They may be able to do so as a result of their capacity to influence not just the PPAR signaling pathway but also the adipocytokine signaling pathway. Both of these separate processes contribute to the production of kidney stones. The logistic regression model displayed better accuracy as well as specificity than individual marker genes when attempting to differentiate kidney stone samples from normal ones.

FZD7 was closely connected to the signaling pathways for focal adhesion, chronic myeloid leukemia, and neurotrophin in a variety of distinct ways. FZD7, which serves as a Wnt receptor in the body, is required for both Wnt signaling and the development of cancer. Researchers have investigated how the several forms of cancer affect the expression of members of the FZD gene family. FZD7 has a function in canonical signaling, and research has demonstrated that colon cancer cells express this gene at a very high level (Ueno et al., 2009). FZD7 is overexpressed in triple-negative breast cancer, according to research by Yang et al. Inhibition of proliferation, invasiveness, and colony formation by FZD7shRNA in MDA-MB-231 and BT-20 cells has been reported (Yang et al., 2011). FZD7 is expressed by the majority of acute lymphoblastic leukemia (ALL) cells (Khan et al., 2007). FZD7 mRNA levels in stage II, stage III, and stage IV tumors were significantly higher than in nontumor tissues in 135 primary colorectal cancer (CRC) tissues. A lower rate of survival was linked to higher FZD7 expression (Ueno et al., 2009). According to Bengochea et al. (2008), FZD7 expression is increased in human hepatocellular carcinoma. By employing quantitative real-time PCR, Janssens et al. investigate the amount of FZDs mRNA expression in more than 30 different human tumor tissues. Renal cell carcinoma (RCC) tissues have been demonstrated to have considerably increased levels of FZD5 and FZD8 mRNA (Janssens et al., 2004). However, FZD7 has not been studied and reported in kidney stone.

GSEA results showed that STK11 was abundant in chemokine signaling pathway, cell adhesion molecules, and antigen processing and presentation. Notably, conditional STK11/LKB1 knockout mice have been shown to develop atypical hyperplasia and prostatic intraepithelial neoplasia (Pearson et al., 2008). STK11 germ line mutations in humans are the cause of Peutz-Jeghers syndrome, a genetic disorder that raises the risk of hamartomas and cancer at various locations such as breast, gastrointestinal and gynecological cancers (Resta et al., 2013). Additionally, sporadic malignancies such endometrial carcinoma, pancreatic, cervical, and non-small-cell lung cancer have been linked to STK11 mutations (Launonen, 2005; Matsumoto et al., 2007; Hezel et al., 2008; Wingo et al., 2009; Contreras et al., 2008; Co et al., 2014). The tumor suppressor serine-threonine kinase STK11 controls a number of biological processes, including cell division, proliferation, cell cycle arrest, differentiation, and cell polarity. These results imply that STK11 may be a key player in human kidney stone development.

Also, results also showed that Type I diabetes was made more active by the downregulation of LCN2. LCN2 was discovered as a tiny protein associated to gelatinase in neutrophils and it was identified as a defensive molecule innately, which has the ability to retain and deplete siderophores that contains iron (Kjeldsen et al., 1993). Recent investigations indicated that the expression level of LCN2 was extensive in critical organs like kidney, heart and brain, and that it might be powerfully activated by inflammatory stimulants (Cowland and Borregaard, 1997; Lu et al., 2009; Ding et al., 2010; Viau et al., 2010). An elevated LCN2 level in the kidney could indicate the severity of renal injury and it is considered to be a well-established marker for chronic renal disorders (Bolignano et al., 2010; Bonomini et al., 2010). In addition to the role as a marker for renal disease, LCN2 is a contributory factor as well. It was found that the chronic renal injury was milder in mice lacking the LCN2 gene (Viau et al., 2010). Lipopolysaccharide-LPS, a bacterial endotoxin, is a powerful inducer of LCN2 (Cowland et al., 2003; Sunil et al., 2007; Moreno-Navarrete et al., 2010). Higher endotoxin levels are associated with both acute and chronic diseases. Multiple organ dysfunctions, including severe renal failure and septic shock, are caused by acute endotoxemia (Smolens and Stein, 1981). On the other hand, chronic endotoxemia linked with obesity, age, and other adverse variables may result in chronic illnesses and persistent inflammatory problems (Moreno-Navarrete et al., 2010; Mehta et al., 2010; Cani et al., 2008). The specific mechanism behind the continuous expression of the LCN2 gene is unclear yet, but LCN2 levels are elevated in both the acute and chronic stages of kidney injury and may be a significant role in the development of chronic kidney disease (Viau et al., 2010). Numerous transcription factors, including AP-1 and C/EBP, are essential for the production of LCN2, as shown by studies using IL-17, IL-1, and other inflammatory stimulants. LPS induces the production of pro-inflammatory mediators via a transient activation of AP-1 (Su et al., 2009). In a separate study (Litvak et al., 2009), it was shown that C/EBP is responsible for the sustained activation of the TLR4 pathway.

The immunological milieu and the production of kidney stones are closely connected, according to a growing body of studies. Using the CIBERSORT method, we compared the immunological microenvironments of individuals with kidney stones to those with normal renal function. Strong positive connections were observed between the FZD7 gene and eosinophils and T gamma delta cells, but negative associations were seen with NK resting cells and CD4^+^ naive T cells. Strong positive association existed between SUV39H1 and M1 macrophages. Using Cytoscape, we were able to predict the effectiveness of 24 different medications that target marker genes, including 23 for STK11 and one for FZD7. Regrettably, we were unable to foresee the emergence of medications that target SUV39H1 and LCN2. We built a ceRNA network by searching the starBase and miRanda databases for data on four marker genes.

In this study, we focused on genes related to ferroptosis and identified possible kidney stone biomarkers using bioinformatics research. We developed a diagnostic tool with a better degree of accuracy as well as specificity and provided information on the prediction of medicines that target marker genes. Furtherly, more studies are needed to get a greater understanding of the mechanisms that lead to the formation of kidney stones and the therapies for their removal.

## Data Availability

Publicly available datasets were analyzed in this study. This data can be found here: https://www.ncbi.nlm.nih.gov/geo/query/acc.cgi?acc=GSE73680.
